# Glucolipotoxicity Impairs Ceramide Flow from the Endoplasmic Reticulum to the Golgi Apparatus in INS-1 β-Cells

**DOI:** 10.1371/journal.pone.0110875

**Published:** 2014-10-28

**Authors:** Enida Gjoni, Loredana Brioschi, Alessandra Cinque, Nicolas Coant, M. Nurul Islam, Carl K. -Y. Ng, Claudia Verderio, Christophe Magnan, Laura Riboni, Paola Viani, Hervé Le Stunff, Paola Giussani

**Affiliations:** 1 Department of Medical Biotechnology and Translational Medicine, Università di Milano, LITA Segrate, Milano, Italy; 2 Unité Biologie Fonctionnelle et Adaptative –UMR CNRS 8251, Université PARIS- DIDEROT (7), Paris, France; 3 School of Biology and Environmental Science and UCD Earth Institute, University College Dublin, Belfield, Ireland; 4 Department of Medical Biotechnology and Translational Medicine, CNR Institute of Neuroscience, Universita' di Milano, Milano, Italy; Louisiana State University Health Sciences Center, United States of America

## Abstract

Accumulating evidence suggests that glucolipotoxicity, arising from the combined actions of elevated glucose and free fatty acid levels, acts as a key pathogenic component in type II diabetes, contributing to β-cell dysfunction and death. Endoplasmic reticulum (ER) stress is among the molecular pathways and regulators involved in these negative effects, and ceramide accumulation due to glucolipotoxicity can be associated with the induction of ER stress. Increased levels of ceramide in ER may be due to enhanced ceramide biosynthesis and/or decreased ceramide utilization. Here, we studied the effect of glucolipotoxic conditions on ceramide traffic in INS-1 cells in order to gain insights into the molecular mechanism(s) of glucolipotoxicity. We showed that glucolipotoxicity inhibited ceramide utilization for complex sphingolipid biosynthesis, thereby reducing the flow of ceramide from the ER to Golgi. Glucolipotoxicity impaired both vesicular- and CERT-mediated ceramide transport through (1) the decreasing of phospho-Akt levels which in turn possibly inhibits vesicular traffic, and (2) the reducing of the amount of active CERT mainly due to a lower protein levels and increased protein phosphorylation to prevent its localization to the Golgi. In conclusion, our findings provide evidence that glucolipotoxicity-induced ceramide overload in the ER, arising from a defect in ceramide trafficking may be a mechanism that contributes to dysfunction and/or death of β-cells exposed to glucolipotoxicity.

## Introduction

Glucolipotoxicity is defined as the condition in which the combined action of elevated glucose and free fatty acid (FFA) levels synergizes in exerting deleterious effects on pancreatic β-cell function and survival [1]–[3]. Accumulating evidence suggests that this condition acts as a key pathogenic component in type II diabetes, contributing to β-cell dysfunction and death during the development of this disease (reviewed in [4]). In agreement, chronic exposure of β-cells to supraphysiological levels of glucose and free fatty acids (FFAs) has been shown to be cytotoxic and cause β-cell dysfunction and failure [5]. Palmitate, a major FFA species in which β-cells might be exposed to *in vivo* [6], is particularly potent in reducing β-cell viability of clonal and primary rodent β-cells, as well as in human islets [7]–[9]. Hyperglycaemia has been shown to potentiate the negative effects of high levels of saturated FFAs on pancreatic β-cells [1], [2]. While palmitate and other saturated FFAs exhibit low toxicity at low glucose concentrations, they have been shown to synergize with elevated glucose concentrations to promote β-cell apoptosis, both in the β-cell line INS-1 and in human islets [10]–[12].

Several mechanisms have been proposed for glucolipotoxicity-induced β-cell dysfunction and failure, and, among them, endoplasmic reticulum (ER) stress and elevations of the proapoptotic sphingolipid ceramide (Cer) appear to play key roles. Additionally, these two processes appear to be strictly connected [13]–[15].

Several enzymes of Cer metabolism have been shown to be involved in regulating its levels in β-cells in response to lipotoxicity and/or glucolipotoxicity. In particular, serine palmitoyltransferase (SPT) and ceramide synthase (CerS), both residing in the endoplasmic reticulum (ER), and involved in Cer biosynthesis [12], [16], [17], as well as neutral sphingomyelinase (N-SMase), involved in Cer degradation [18], [19], have emerged as important regulators of elevated Cer levels. In addition, the over-expression of glucosyl-ceramide synthase, which converts Cer into glucosyl-ceramide (GlcCer), has been shown to prevent β-cell apoptosis [20]. Altogether these data suggest that the accumulation of Cer in the ER compartment of β-cells is crucial in determining β-cell fate, *i.e.*, survival or death.

While the accumulation of Cer at the ER consequent to glucolipotoxicity appears to be critically involved in the induction of ER stress (reviewed in [15]), the dysregulation of Cer metabolism alone may not necessarily lead to Cer accumulation in the ER unless Cer traffic from the ER to the Golgi is inhibited. Cer synthesized in the ER is transferred to the Golgi where it is subsequently converted to sphingomyelin (SM), GlcCer and more complex glycosphingolipids (GSLs) [21]. Evidence to date indicates that there are two pathways by which Cer is transported from the ER to the Golgi: a protein-mediated transport, by the soluble ceramide transfer protein CERT (for SM formation) [22]–[25], and a CERT-independent vesicular traffic (for the biosynthesis of SM or GlcCer) [23], [25], [26]. The two modes of Cer transport coexist separately contributing to the regulation of Cer metabolism and levels in cells. For example, hyperphosphorylation of a serine repeat motif of CERT impairs its binding to the ER and Golgi membranes, thereby inhibiting Cer transfer from the ER to the Golgi [22], [27], [28]. Additionally, nitric oxide or the overexpression of sphingosine-1-phosphate phosphohydrolase 1 (SPP1) inhibits Cer vesicular traffic [26], [29], resulting in Cer accumulation in the ER. Notwithstanding, our knowledge on the effect of glucolipotoxicity on Cer transport is scarce.

The aim of this investigation was to determine if the transport mechanisms of Cer from the ER to the Golgi are involved in the deleterious effects of glucolipotoxicity in β-cells, and to gain a further understanding of the relationship between Cer accumulation and ER stress. We demonstrate, using INS-1 cells as a model, which can be expanded to quantities sufficient for diverse experimentation, that palmitate and elevated glucose administration induced a rapid and potent inhibitory effect on the mechanisms of Cer transport, resulting in the accumulation of Cer at the ER.

## Material and Methods

### Materials

All reagents were of analytical grade unless otherwise stated. The tissue culture medium RPMI 1640 was purchased from Lonza (Basel, Switzerland). L-glutamine, sodium pyruvate solution, penicillin/streptomycin, dimethyl sulfoxide (DMSO), palmitate, glucose, Hepes, bovine serum albumin fraction V (BSA), fatty acid free-BSA, 3-[4,5-dimethylthiazol-2-yl]2,5-diphenyl tetrazolium bromide (MTT), leupeptin, aprotinin, wortmannin (Wm), Thapsigargin (Tg), Kodak Biomax film, HPLC grade water, tetrahydrofuran (THF), methanol, LC-MS grade water, formic acid and ammonium formate were purchased from Sigma-Aldrich (St. Louis, MO, USA). Fetal calf serum (FCS) was from Euroclone (Pero, Milano, Italy). LY294002 was from Cayman Chemical (Ann Arbor, MI, USA). Lipofectamine 2000 and the Stealth RNAi were from Invitrogen (Carlsbad, CA, USA). D-erythro-[3-^3^H]sphingosine (Sph) (19.7 Ci/mmol), was from PerkinElmer Life Science (Boston, MA, USA). Pepstatin was from Roche Applied Sciences (Mannheim, Germany). High performance thin layer chromatography (HPTLC) silica gel plates were from Merck (Darmstadt, Germany). The Golgi marker Texas red wheat germ agglutinin (WGA), 6-((N-(7-nitrobenz-2-oxa-1,3-diazol-4-yl) amino) hexanoyl) sphingosine (NBD-C_6_Cer) and N-(4,4,-difluoro-5-,7-dimethyl-bora-3a,4a-diaza-sindacene- 3-pentanoyl) sphingosine (BODIPY-C_5_Cer) were from Life Technologies (Italy). The antibodies recognizing Phospho-Akt (Ser473) were from Cell Signaling Technology, Inc. (Danvers, MA, USA); polyclonal antibodies against Cer transfer protein (CERT) from Bethyl Laboratories (Montgomery, TX, USA). Primary mouse monoclonal anti-phospho-serine, goat anti-GRP78 and rabbit anti-GAPDH antibodies, and secondary HRP-conjugated anti-rabbit or anti-goat antibodies were from Santa Cruz Biotechnology (Santa Cruz, CA, USA). Secondary anti-mouse HRP-conjugated antibody, SuperSignal WestPico Chemioluminescent Substrate and SuperSignal WestFemto Maximum Sensitivity Substrate were from Thermo Scientific (Rockford, IL, USA). Ceramide/Sphingoid Internal Standard Mixture I from Avanti Polar Lipids (Alabaster, Alabama, USA) was used for quantitative analysis. The plasmid of CERT tagged with green fluorescent protein (CERT-GFP) was kindly provided by Dr. Maria Antonietta De Matteis, Telethon Institute of Genetics and Medicine, Napoli (Italy).

### Cell culture conditions

Rat insulinoma INS-1 cells, kindly provided by Merck–Serono, were grown in RPMI 1640 medium buffered with 10 mM Hepes containing 10% (v/v) FCS, 2 mM L-glutamine, 1 mM sodium pyruvate, 50 µM 2-mercaptoethanol and 100 units/ml penicillin/streptomycin at 37°C in an atmosphere of 5% CO_2_ and 95% humidified air. Before each experiment, INS-1cells plated at 2×10^5^ cell/cm^2^ were cultured for 24 h in RPMI 1640 plus 10% FCS. Cells were then cultured in the presence of 5 mM or 30 mM glucose with or without 0.4 mM palmitate for 12 h; incubation in the presence of 30 mM glucose and 0.4 mM palmitate mimics glucolipotoxicity conditions. When indicated, the cells were preincubated with 20 µM LY294002, or 10 nM Wm or 0.1 µM Tg for 30 min. Palmitate was administered to the cells as a conjugate with fatty-acid-free BSA. Briefly, dried aliquots of palmitate in ethanol were dissolved in PBS containing 5% (w/v) BSA to obtain a 4 mM stock solution. The molar ratio of FFAs to BSA was 5∶1. The FFA stock solutions were diluted in RPMI 1640 medium supplemented with 1% FCS to obtain a 0.4 mM final concentration at a fixed concentration of 0.5% BSA.

### Analysis of cell viability

Cell viability was determined by MTT assay. INS-1 cells were plated and grown on a glass coverslip and cultured in the presence of 5 mM or 30 mM glucose with or without 0.4 mM palmitate for 12 h, as previously described, or for 24 hours. At the end of the treatments, the medium was replaced by MTT dissolved in fresh medium (0.8 mg/ml) for 4 hours. The formazan crystals were then solubilized in isopropanol/formic acid (95∶5 v/v) for 10 minutes and the absorbance (570 nm) was measured using a microplate reader (Wallack Multilabel Counter, Perkin Elmer, Boston, MA, USA).

### RNA interference

Small interfering RNA (siRNA) duplexes for rat CERT (Gene accession number XM 345143.1) S87, S522 and control non-targeting siRNAs (scrambled sequences of S87 and S522 oligonucleotides) described in [23] were used. We used Stealth RNAi, the chemically modified synthetic RNAi duplexes that virtually eliminate the induction of non-specific cellular stress response, and that also improve the specific, effective knock-down of gene expression. INS-1 cells plated at 2×10^5^ cell/cm^2^ were maintained for 24 h in RPMI 1640 plus 10% FCS and then transfected in the same medium with a 1∶1 (by mol) mix of S87 + S522 (si-CERT) or the non-targeting corresponding sequences (si-CT) using LipofectAMINE 2000 according to the manufacturer's protocol. The final concentration of siRNA–lipofectamine duplex mixture was 100 nM. All the experiments were performed 72 h after transfection.

### Plasmid Transfection

INS-1 cells were plated at 2×10^5^ cell/cm^2^ on a glass coverslip and grown in RPMI 1640 supplemented with 10% FCS until they were 50–70% confluent. Then cells were transfected with expression plasmid encoding the protein CERT tagged with GFP (CERT-GFP) or pcDNA3.1 empty vector using the Lipofectamine 2000 reagent according to the manufacturer's directions.

### [^3^H]Sphingosine metabolism

INS-1 cells, si-control (siCT) and si-CERT (siCERT) INS-1 cells cultured in the presence of 5 mM or 30 mM glucose with or without 0.4 mM palmitate for 12 h were pulsed with [^3^H]Sph (0.3 µCi/ml), for 1 h maintaining the treatment conditions. All experiments were performed at 37°C. Stock solutions of [^3^H]Sph in absolute ethanol were prepared and added to fresh medium. In all cases, the final concentration of ethanol never exceeded 0.1% (v/v). At the end of pulse, cells were washed twice with phosphate-buffered saline (PBS) at 4°C, harvested and submitted to lipid extraction and partitioning as previously described [30]. The methanolized organic phase and the aqueous phase were analyzed by HPTLC using chloroform/methanol/water (55∶20∶3 by vol) and chloroform/methanol/0.2% CaCl_2_ (55∶45∶10 by vol) as solvent system respectively. Digital autoradiography of HPTLC plates was performed with Beta-Imager 2000 (Biospace, France) and the radioactivity associated with individual lipids was measured using the software provided with the instrument. The [^3^H]-labeled sphingolipids were recognized and identified as previously described [30].

### Liquid chromatography-tandem mass spectrometry (LC-MS/MS) protocol for lipid extraction and quantitation

Cellular lipids were extracted from INS-1 cells according to Shaner *et al.* [31] with modifications. Briefly, freeze-dried INS-1 cells (2 million) were transferred to a 5 ml glass tube, spiked with 10 µl of internal standard (12.5 µM Ceramide/Sphingoid Internal Mix I), and extracted with 2 ml of chloroform∶methanol (1∶2, v/v) following brief sonication and constant agitation in a 50°C water bath for 2 h. After cooling to room temperature, 200 µl of 1 M KOH in methanol was added and incubated for 2 h at 37°C. After cooling, 15 µl of glacial acetic acid were added to neutralize the extract. Following centrifugation, the supernatant was transferred to a new glass tube and the extraction was repeated with a further 2 ml of chloroform∶methanol (1∶2, v/v). The resulting supernatants were pooled and dried under a stream of N_2_. The lipids were resuspended in 200 µl of mobile phase (mobile phase A: mobile phase B, 1∶1, v/v). After centrifugation, aliquots were used for LC-MS analysis. Inorganic phosphate (Pi) content of extracts were determined according to van Veldhoven and Mannaerts [32]. Peak areas were used for quantitation by comparison with the peak areas of internal standards.

Chromatographic separation of lipids was performed with an HPLC system consisting of a binary pump, auto sampler, column oven (1200 RRLC, Agilent Technologies, http://www.chem.agilent.com). The lipid molecules were separated using a GeminiNX C_18_ analytical column (2.0 mm I.D. x 100 mm, particle size 3 µm, Phenomenex, http://www.phenomenex.com). Column oven and auto sampler temperatures were maintained at 45°C and 4°C, respectively. The mobile phase consists of solvent A (15 mM ammonium formate (pH 4.0):MeOH∶THF, 5∶2∶3) and solvent B (15 mM ammonium formate (pH 4.0):MeOH∶THF, 1∶2∶7). Elution was performed at a flow rate of 0.30 ml min^-1^ in a binary gradient mode. The initial composition of mobile phase was 65∶35 (A∶B), linearly changed to 70∶30 (A∶B) over 12 min, and maintained this composition over 22 min, changed to initial composition 65∶35 (A∶B) over 1 min, followed by 7 min of column re-equilibration. Column eluant was directed to waste for the initial 1 min.

The HPLC system was coupled online to an Agilent 6460 triple quadrupole mass spectrometer (Agilent Technologies) equipped with a Jet Stream ion source. Data were recorded in positive ionization mode using electrospray ionization with nitrogen as the nebulizing gas. The gas temperature and flow rate was 350°C and 10 l min^−1^, and the sheath gas temperature and flow rate was 360°C and 12 l min^−1^, respectively. The ESI needle voltage was adjusted to 4000 V in positive mode and optimum fragmentor voltages and collision energies were assigned by analysis of reference compounds (SM (d18∶0/12∶0); Cer (d18∶1/12∶0); GlcCer (d18∶1/12∶0); S1P (d18∶1-P)) in selected ion and product ion scanning mode to determine multiple-reaction monitoring (MRM) conditions and mass spectrometric structural studies. MRM detection was applied using nitrogen as the collision gas.

### Analysis of the Intracellular Distribution of Fluorescent Ceramides

INS-1 cells were plated and grown on a glass coverslip and cultured as previously described. At the end of the treatments, the cells were loaded with 2.5 µM BODIPY-C_5_-Cer or NBD-C_6_-Cer (as 1∶1 complex with fatty acid free BSA) in RPMI 1640 at 4°C for 30 min [23]. After loading, the cells were incubated 30 min at 37°C in RPMI 1640 containing 5 mM or 30 mM glucose ± 0.4 mM palmitate and fixed with 0.5% glutaraldehyde solution in PBS for 10 min at 4°C. The specimens were immediately observed and analyzed with a fluorescence microscope (Olympus BX-50) equipped with a fast high resolution charge-coupled device camera (Colorview 12) and an image analytical software (Analysis from Soft Imaging System GmbH).

### Analysis of Intracellular Localization of CERT-GFP by Confocal microscopy

INS-1 cells plated at 2×10^5^ cell/cm^2^ were grown on a glass coverslip and maintained 24 h in RPMI 1640 plus 10% FCS. The cells were then transfected with the plasmid, CERT-GFP using lipofectamine 2000 according to manufacturer's instructions. 24 h after transfection, cells were treated with 5 mM or 30 mM glucose ±0.4 mM palmitate for 12 h and fixed with 0.5% glutaraldehyde solution in PBS for 10 min at 4°C. The cells were then permeabilized with 0.2% Triton X-100 for 30 min at room temperature and stained with WGA-texas red. The specimens were analyzed with a confocal microscope (Leica SP5).

### Immunoblotting

Phosho-Akt and GRP78 immunoblotting were performed on INS-1 cells lysed with lysis buffer (20 mM Tris-HCl pH 7.4, 150 mM NaCl, 1% NP-40, 10 mM sodium fluoride, 1 mM EDTA, 10 mM Na_4_P_2_O_7_, 1 mM Na_3_VO_4_, and the protease inhibitor cocktail). Solubilized proteins were centrifuged at 14,000×g at 4°C for 10 min. Supernatants were subjected to 10% SDS polyacrylamide gel electrophoresis and transferred to nitrocellulose membranes. Membranes were blocked for 1 h at room temperature in Tris-buffered saline (10 mM Tris-HCl, pH 7.4, 140 mM NaCl) containing 0.1% Tween-20 (TBS-T) and 5% skim milk, and then incubated with primary antibodies against phosho-Akt overnight at 4°C or against GRP78 1 h at room temperature. Membranes were washed in TBS-T, and bound antibodies visualized with horseradish peroxidase-coupled secondary antibodies (Santa Cruz Biotechnology) and chemiluminescent substrate. The relative intensities of bands were quantified by densitometry.

CERT immunoblotting were performed using wild type or si-control and si-CERT transfected cells lysed with CERT buffer (10 mM Tris-HCl pH 7.4, 0.25 mM sucrose, 0.5 mM phenylmethylsulfonyl fluoride, 10 µg/ml aprotinin, 5 µg/mL leupeptin, 5 µg/mL pepstatin), processed and analyzed as previously described [23]. The membranes were stripped 30 minutes at 50°C in 2% SDS, 100 mM DTT, 0.5 M Tris-HCl pH 6.8, washed in TBS-T and incubated 1 hour in Tris-buffered saline (10 mM Tris-HCl, pH 7.4, 140 mM NaCl) containing 0.1% Tween-20 (TBS-T) and 5% BSA and then incubated with the primary antibody against phospho-serine 2 h at room temperature. Membranes were washed in TBS-T and bound antibodies visualized with horseradish peroxidase-coupled secondary antibodies (Santa Cruz Biotechnology) and chemiluminescent substrate.

### RNA isolation, reverse transcription and Real-Time PCR

INS-1 cells were plated and grown on a glass coverslip and cultured as previously described. At the end of the treatments, total RNA was isolated from INS-1 cells with the RNeasy mini kit and treated with the RNase-free DNAse I. One microgram of RNA was reverse transcribed using the iScript cDNA synthesis kit according to manufacturer's instructions. Real-Time PCR was performed using the iQ5 Real-Time PCR detection system (Biorad Laboratories, Hercules, CA, USA). Specific SYBR green expression assays (SYBR green super mix) for CERT and TBP (TATA-box-binding protein) were carried out. Simultaneous amplification of the target sequences was carried out as follows: 3 minutes at 95°C, 50 cycles 95°C 10 sec, 59°C 40 sec and 60°C 30 sec and 1 cycle of 60°C 3 minutes. Results were analyzed using the iQ5 optical system software (Biorad Laboratories, Hercules, CA, USA). Relative gene expression was determined using the 2^−ΔΔ*C*t^ method [33]. Data were normalized to TBP expression (used as endogenous control) and INS-1 G5 cells were used as calibrator.

### Sphingomyelin synthase activity

INS-1 cells were plated and treated as described above. At the end of the treatments, the cells were loaded with 2.5 µM NBD-C_6_-Cer (as 1∶1 complex with fatty acid free BSA) in RPMI 1640 at 4°C for 30 min. After loading, the cells were incubated 15 or 30 min at 37°C in RPMI 1640 with 5 mM glucose or 30 mM glucose ±0.4 mM palmitate. At the end of the incubation, cells were immediately put at 4°C to stop the enzymatic reaction; lipids were extracted with chloroform -methanol [34] and separated by thin-layer chromatography (TLC) using chloroform/methanol/0.1 M KCl (1∶2∶0.8 [vol/vol/vol]) as the developing solvent. Fluorescence-labeled sphingomyelin was quantified with a luminescence spectrometer (LS50B PerkinElmer).

### Other methods

Total protein amount was assayed with the Comassie Blue based Pierce reagent, using BSA fraction V as standard. Radioactivity was measured by liquid scintillation counting.

### Statistical analysis

Statistical significance of differences was determined by one-way ANOVA.

## Results

### Effect of palmitate and glucose on [^3^H]Sph metabolism in INS-1 cells

**Figure 1:**
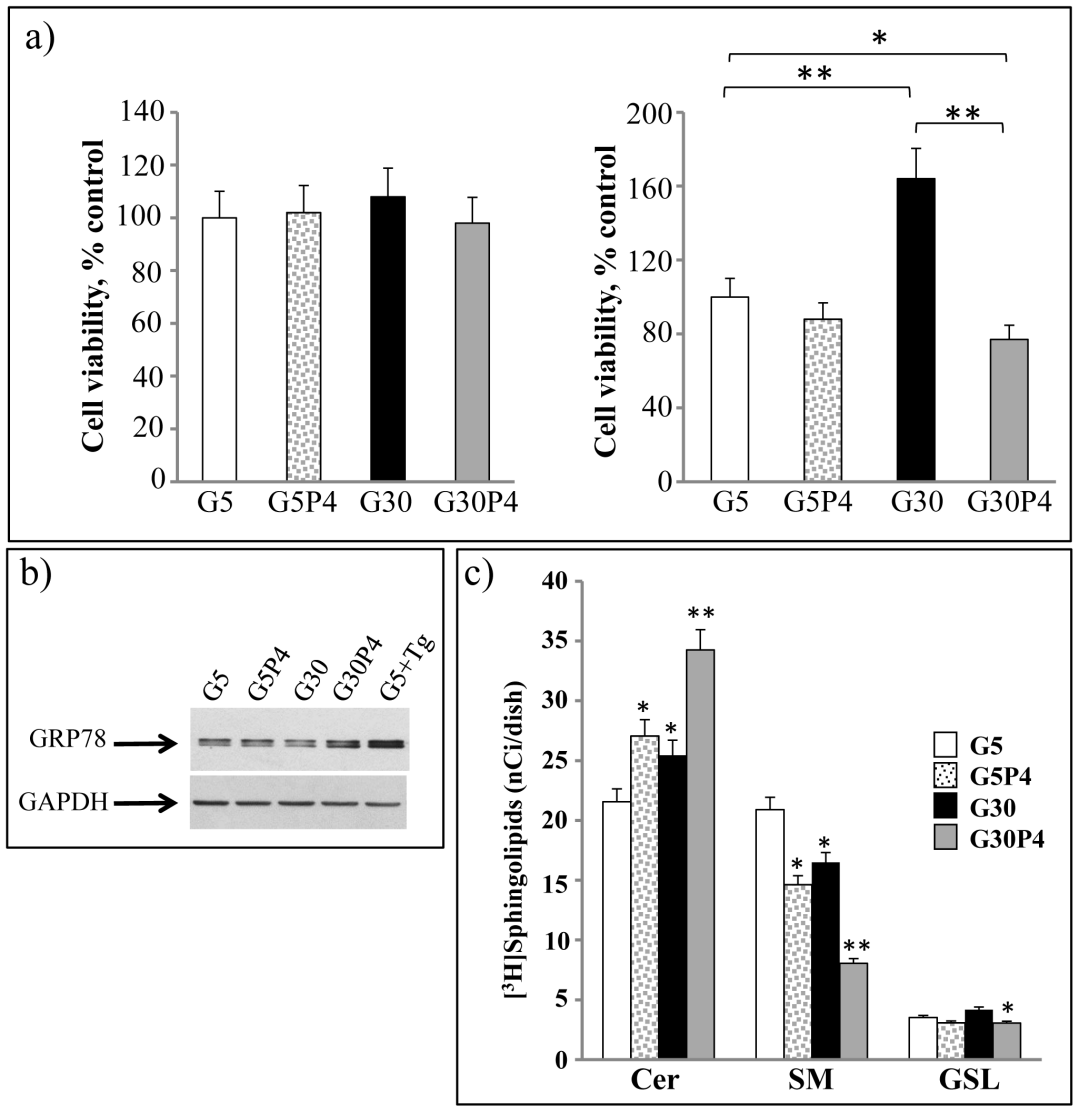
Palmitate and glucose regulate the use of Cer for the biosynthesis of complex sphingolipids in INS-1 cells. a) Cells were treated for 12 h (left panel) or 24 h (right panel) with 0.4 mM palmitate (P4) or without palmitate in the presence of 5 mM or 30 mM glucose. Cell viability was assessed by the MTT assay. Results are expressed as percentage of cell viability with respect to 5 mM glucose-treated cells (100%). Data are the mean ± S.D. of three independent experiments. *, p<0.05; ** p, <0.01. b) INS-1 cells were treated with 5 mM or with 30 mM glucose ±0.4 mM palmitate and harvested in lysis buffer for immunoblot analysis of GRP78 and GAPDH levels as described in experimental procedures. INS-1 cells were pretreated 30 min ±0.1 µM thapsigargin (Tg). Equal amounts of protein from homogenates were analyzed by immunoblotting with an anti-GRP78 antibody and an anti-GAPDH antibody. c) Cells were treated for 12 h ±0.4 mM palmitate in the presence of 5 mM or 30 mM glucose and then pulsed with 0.3 µCi/ml [C3-^3^H]sphingosine for 1 h. At the end of pulse, cells were harvested and submitted to lipid extraction and partitioning. The methanolized organic phase and the aqueous phase were analyzed by HPTLC and digital autoradiography of HPTLC (see experimental procedures). G5, 5 mM glucose; G5P4, 5 mM glucose+0.4 mM palmitate; G30, 30 mM glucose; G30P4, 30 mM glucose+0.4 mM palmitate. Data are the mean ± S.D. of at least three independent experiments. *p<0.05 for Ceramide and Sphingomyelin G5P4 or G30 compared with G5 and for GSLs G30P4 compared with G30; **p <0.01 for Cer and SM G30P4 compared with G30.

Treatment for 12 h with 0.4 mM palmitate (G5P4), 30 mM glucose (G30), or 0.4 mM palmitate plus 30 mM glucose (G30P4) did not exert a toxic effect on INS-1 control cells treated with 5 mM glucose (G5) (Fig. 1a, left panel). A similar effect of palmitate on cell viability was observed when assessing the protein content of each dish, demonstrating that at 12 hours there is no toxic effect on INS-1 cells (results not shown). After 24 hours, palmitate or 30 mM glucose alone exerted no toxicity. We also observed that 30 mM glucose increased INS-1 cell numbers, in agreement with previously published report [12] (Fig. 1a). In contrast, co-administration of 0.4 mM palmitate and 30 mM glucose for 24 hours reduced INS-1 cell viability by 53% (Fig. 1a right panel). Western blot analysis of GRP78, a ER stress marker, showed that a 12 h co-treatment with 0.4 mM palmitate and 30 mM glucose induced about 60% increase in the amount of GRP78 compared to 5 mM glucose with or without 0.4 mM palmitate and 30 mM glucose in INS-1 cells (Fig. 1b), suggesting that glucolipotoxic conditions induce ER stress. In the positive control, we observed that 0.1 µM thapsigargin in the presence of 5 mM glucose doubled GRP78 levels in INS-1 cells (Fig. 1b). To evaluate the effects of palmitate and high glucose concentrations on Cer utilization for the biosynthesis of SM and GSLs in INS-1 cells, we studied Cer metabolism using [^3^H]-sphingosine as a metabolic precursor as it is rapidly internalized in the cells and efficiently N-acylated to Cer, which in turn is converted to SM, glucosylceramide and complex GSLs. We performed short pulse experiments to monitor the utilization of newly synthesized Cer for the biosynthesis of SM and GlcCer [26]. In all cases, [^3^H]Sph was mainly metabolized to N-acylated compounds, mostly represented by Cer, SM and, in lower amounts, GSLs (Fig. 1c); the extent of N-acylation being similar in all conditions. Treatment with palmitate in the presence of 5 mM glucose significantly modified the distribution of radioactivity between the different Sph metabolites. Our results showed that palmitate induced [^3^H]Cer accumulation was associated with a decrease of [^3^H]SM levels (Fig. 1c). Treatment with 30 mM glucose by itself also reduced [^3^H]Cer conversion to [^3^H]SM. Moreover, high glucose levels strongly potentiated the reduced utilization of Cer for SM biosynthesis induced by 0.4 mM palmitate, and also significantly reduced GSL biosynthesis (Fig. 1c). The percent increase of [^3^H]Cer was 23% and 14%, respectively in 5 mM glucose plus palmitate, and in 30 mM glucose treated cells; in cells treated with 30 mM glucose plus palmitate [^3^H]Cer was 54% higher (Fig. 1c) than in cells treated with 5 mM glucose. Conversely, the reduction in [^3^H]SM was 32% and 23% in cells treated separately with 0.4 mM palmitate and 30 mM glucose, respectively, and 64% in glucolipotoxic conditions, demonstrating that co-treatment with 30 mM glucose and palmitate has a greater effect than the sum of the individual treatments (palmitate or 30 mM glucose) (Fig. 1c). Taken together, these results showed that in pancreatic β-cells, glucolipotoxicity can regulate the use of Cer for the biosynthesis of complex sphingolipids in the Golgi apparatus.

### Effect of palmitate and glucose on ceramide, sphingomyelin and glucosylceramide molecular species

**Figure 2:**
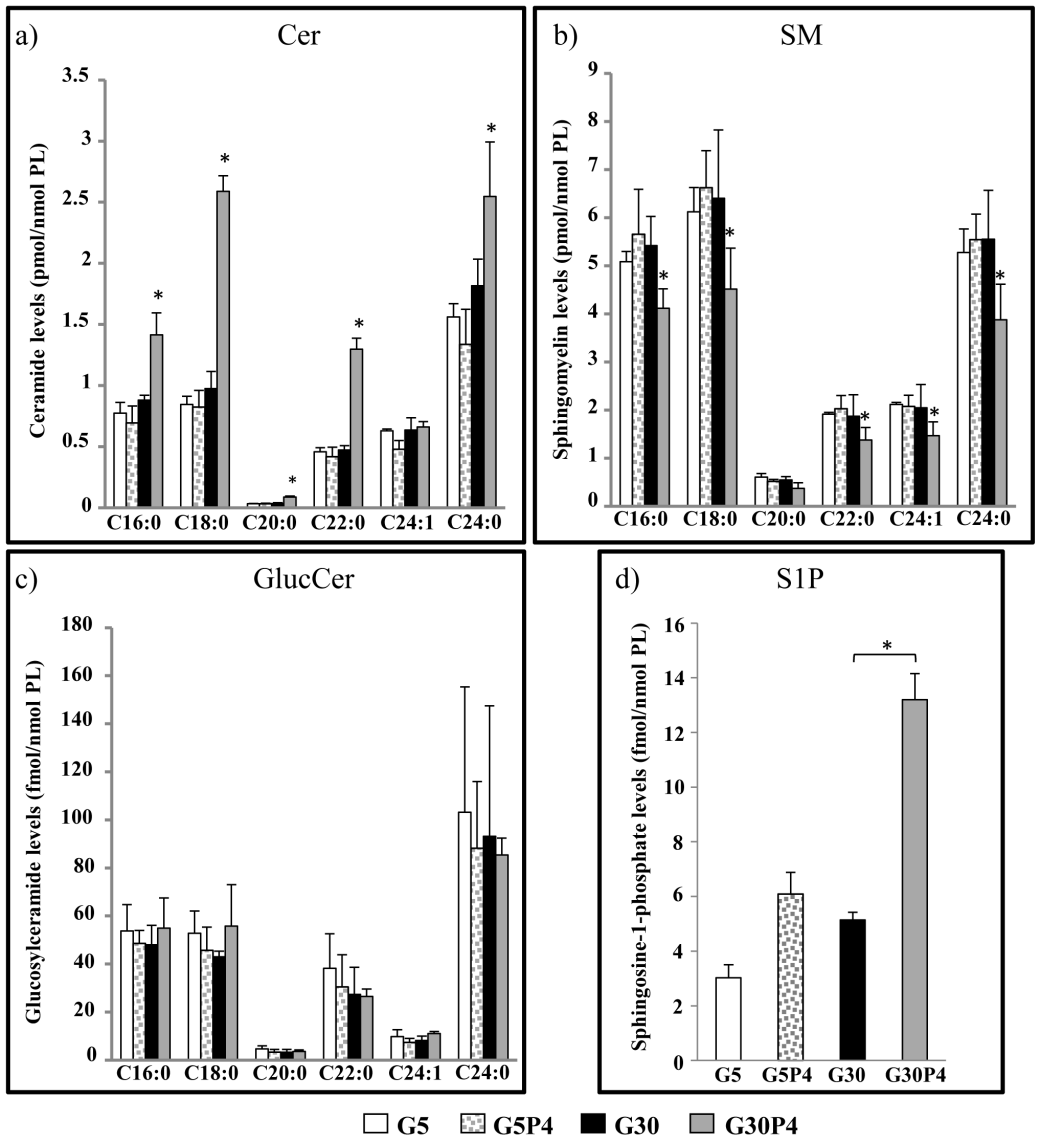
Chain-length specificity of ceramide, sphingomyelin and glucosylceramide in response to palmitate and high concentrations of glucose in INS-1 cells. Cells were incubated with 0.4 mM palmitate in the presence of 5 mM (G5) or 30 mM (G30) glucose for 12 h. Levels of N-acyl chain lengths of Cer, SM and GlcCer were determined by LC–MS/MS. Levels of S1P in INS-1 cells were also determined by LC-MS/MS measurement. Results are expressed as pmol/nmol of phospholipids (PL) for Cer and SM and as fmol/nmol PL for GlcCer and S1P and are means ± S.D. for three independent experiments. *p<0.05 vs G5 except for S1P *p<0.05 vs G30.

Next, we evaluated the effect of palmitate treatment on Cer, SM and GlcCer mass levels, and the levels of their metabolic molecular species by LC/MS/MS. Our data showed that palmitate, in the presence of 5 mM glucose as well as 30 mM glucose alone, did not significantly alter Cer, SM and GlcCer mass levels in INS-1 cells (Fig. 2); In contrast, palmitate with high glucose levels (30 mM) promoted an increase in Cer mass levels with a concomitant decrease in the mass levels of SM but not that of GlcCer. Our data show that glucolipotoxic conditions led to an increase in saturated ceramides, the greatest increase being observed in C18∶0-Cer and C22∶0-Cer (Fig. 2). In addition, this condition mostly reduced the levels of saturated SM and, more specifically, of C18∶0-SM and C24∶0-SM (Fig. 2). In contrast, glucolipotoxicity did not appear to alter significantly the levels of the different GlcCer molecular species (Fig. 2). We also evaluated the effect of palmitate treatment on S1P levels in INS-1 cells. Our data showed that at low levels of glucose (5 mM), palmitate was unable to increase significantly S1P levels in INS-1 cells. However, after a 12 h of treatment, palmitate in the presence of 30 mM glucose promoted an increase in S1P levels (Fig. 2) in agreement with the findings of Verét et al. [35]. Altogether, these data suggest that glucolipotoxicity induced an increase in Cer levels and a reduction of SM levels in pancreatic β-cells, but did not appear to significantly affect the amounts of GlcCer.

### Effect of palmitate and glucose on Sphingomyelin Synthase activity

We then evaluated if the decrease in SM levels induced by glucolipotoxicity is due to the inhibition of SM synthase (SMS) activity using NBD-C_6_-Cer, a fluorescently labeled ceramide that is an efficient substrate for SM synthases. Treatment with palmitate in the presence of different glucose concentrations was unable to alter the activity of SMS in INS-1 cells (data not shown), suggesting that the activities of enzymes responsible for the biosynthesis of SM were not affected by glucolipotoxic conditions in INS-1 cells.

### Effect of palmitate and glucose on intracellular distribution of BODIPY-C_5_Cer and NBD-C_6_Cer

**Figure 3:**
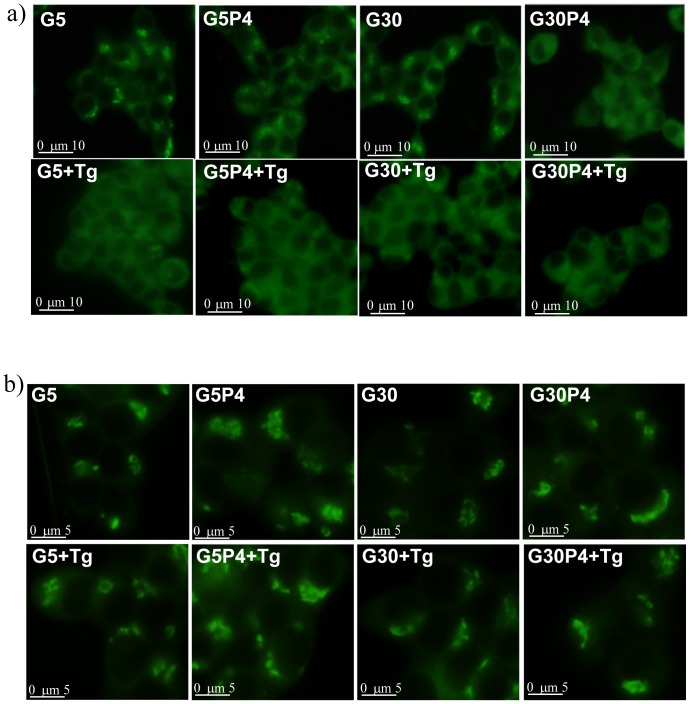
Palmitate and glucose impairs ceramide flow from the ER to the Golgi apparatus in INS-1 cells. INS-1 cells grown on a glass coverslip were pretreated 30 min ± 0.1 µM thapsigargin. At the end of the pretreatment, the cells were treated with or without palmitate in the presence of 5 mM or 30 mM glucose for 12 h and then incubated with a) 2.5 µM BODIPY-C_5_Cer or b) 2.5 µM NBD-C_6_Cer as BSA complex 1∶1 (m/m) in DMEM for 30 min at 4°C; labeled cells were incubated at 37°C for 30 min and analyzed. All images were processed and printed identically.

We then tested the possibility that glucolipotoxicity inhibited the synthesis of complex sphingolipids, mainly represented by SM, by affecting the transport of Cer synthesized in the ER to the Golgi apparatus (where SM and GSL biosynthesis occurs). The transport of natural Cer from the ER to the Golgi apparatus can be qualitatively evaluated from the analysis of BODIPY-C_5_-Cer redistribution in cells [34]. In 5 mM and 30 mM glucose-treated INS-1 cells, most of the fluorescence accumulated in the perinuclear region (Fig. 3a), which is representative of the Golgi apparatus. In INS-1 cells treated with 0.4 mM palmitate together with 5 mM glucose, fluorescence was also observed in the perinuclear region but to a lesser extent compared to control cells suggesting a partial defect in Cer traffic. (Fig. 3a). In contrast, co-administration of 0.4 mM palmitate and 30 mM glucose strongly reduced fluorescence accumulation in the Golgi apparatus region (Fig. 3a), suggesting an impairment of ceramide flow from the ER to the Golgi apparatus as a result of glucolipotoxicity in pancreatic β-cells. In INS-1 cells, the presence of thapsigargin (Tg) which induced ER stress in β-cells (Fig. 1b) [14] mimics the effect of high glucose together with 0.4 mM palmitate by strongly reducing the fluorescence accumulation in the Golgi apparatus region (Fig. 3a). The effect of thapsigargin was not affected by the presence of glucose or palmitate. In contrast, when cells were labeled with NBD-C_6_Cer, which selectively localizes at the Golgi apparatus [34], 5 mM and 30 mM glucose in the presence or absence of 0.4 mM palmitate with or without Tg did not modify the accumulation of NBD fluorescence in the perinuclear Golgi region (Fig. 3b). Altogether, these results suggest that glucolipotoxicity induce an impairment of ceramide flow from the ER to the Golgi apparatus in pancreatic β-cells.

### Effect of palmitate and glucose on CERT levels and activation

**Figure 4:**
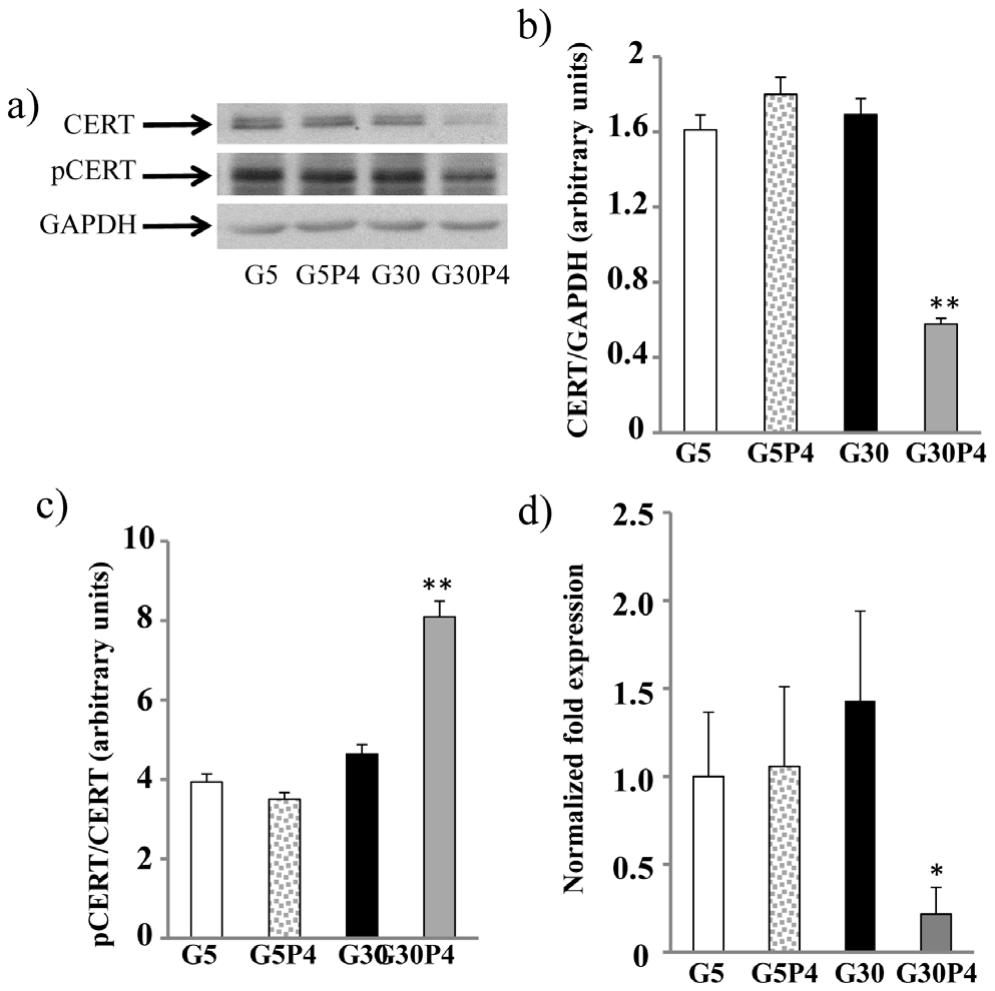
Palmitate and glucose regulate CERT expression and activation in INS-1 cells. a) INS-1 cells were harvested in lysis buffer as described in material and methods. Equal amounts of protein from homogenates were analyzed by immunoblotting with an anti-CERT antibody, an anti-phosphoserine and an anti-GAPDH antibody; b) the amount of CERT expressed was determined by densitometric quantitation and normalized for GAPDH **p<0.01 for G30+palmitate compared with G30; c) the amount of pCERT expressed was determined by densitometric quantitation and normalized for CERT **p<0.01 for G30+palmitate compared with G30; d) Relative expression of CERT assessed by Real-Time PCR. Results are expressed as fold-change relative to G5 *p<0.05 G30+palmitate cells vs G30. Values are mean ± SD of three independent experiments.

A recent report suggests that inhibition of CERT-mediated Cer transport can exacerbate repression of pro-insulin gene expression induced by long term treatment (48 h) with palmitate in INS-1 cells [36]. On this basis, we evaluated if a shorter treatment (12 h) of INS-1 cells with palmitate and glucose can affect the levels and the activation status of the protein CERT. Western blot analysis showed that both 0.4 mM palmitate or 30 mM glucose alone did not significantly alter the total amount of the CERT protein (Fig. 4a and b) whereas co-treatment with 0.4 mM palmitate and 30 mM glucose induced a 65% reduction in the total amount of CERT in INS-1 cells (Fig. 4a and b). Moreover, using an antibody against phospho-serine according to Guo and co-workers [36], we found that palmitate or 30 mM glucose alone did not modify significantly the amount of phospho-CERT. Interestingly, co-administration of 0.4 mM palmitate and 30 mM glucose doubled the levels of phospho-CERT in INS-1 cells (Fig. 4a and c). In order to assess how palmitate plus high glucose regulates CERT levels, CERT expression was evaluated by quantitative PCR in INS-1 cells. The results obtained (Fig. 4d) show that palmitate in the presence of 30 mM glucose induced a 80% reduction in steady-state levels of CERT transcripts, thus demonstrating that glucolipotoxicity regulates CERT expression by inhibiting its synthesis.

### Effect of palmitate and glucose on CERT subcellular localization

**Figure 5:**
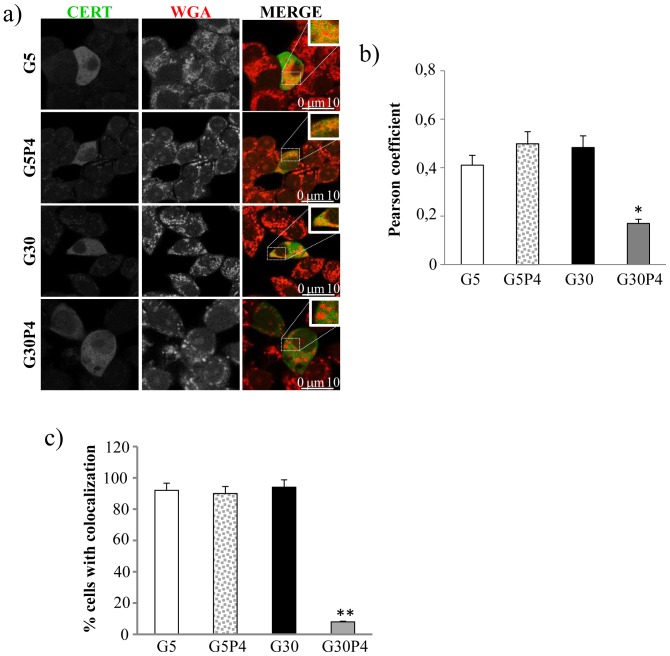
Palmitate and glucose prevent colocalization of CERT and Golgi apparatus in INS-1 cells. INS-1 cells grown on a glass coverslip were transfected with the plasmid CERT-GFP as described in experimental procedures. 24 h later, the cells were treated with or without palmitate in the presence of 5 mM or 30 mM glucose for 12 h. Cells were then fixed and immunostained with WGA texas red-conjugated, a specific marker for the Golgi apparatus. a) Representative confocal microscopy images are shown; all images were processed and printed identically. b) The co-localization between CERT and WGA has been quantified through the Image J software and reported as Pearson colocalization coefficient. *p<0.05 G30+palmitate cells vs G30. c) The percentage of cells with co-localization of CERT and WGA was determined. The data are means ± the SD. **p<0.01 for G30+palmitate compared with G30.

The phosphorylated form of CERT should have the PH domain, the docking site for the Golgi apparatus, covered by the START domain and should therefore not be able to colocalize with the Golgi apparatus [37]. With this premise, we evaluated the ability of CERT to localize at the Golgi apparatus in INS-1 cells as a measure of CERT activity. To this purpose, we analysed the co-localization of over-expressed CERT-GFP with the Golgi marker WGA in INS-1 cells. The images obtained (Fig. 5a) showed that CERT and WGA co-localized in INS-1 cells treated with 5 mM glucose in the presence or the absence of 0.4 mM palmitate, or treated with 30 mM glucose. In contrast, co-treatment with 30 mM glucose and 0.4 mM palmitate significantly reduced co-localization between CERT and WGA in INS-1 cells (Fig. 5a) according to the Pearson's colocalization coefficients (Fig. 5b). Moreover when INS-1 cells were treated with 5 mM glucose in the presence or absence of 0.4 mM palmitate, or treated with 30 mM glucose alone, CERT and WGA co-localize in more than 90% of the cells analysed (Fig. 5c). In contrast, in cells treated with 30 mM glucose in the presence of 0.4 mM palmitate, co-localization of CERT and WGA was detectable in only 8% of the cells (Fig. 5c). Altogether, our results (Fig. 4 and 5) demonstrated that glucolipotoxicity can impair CERT-mediated Cer transport by reducing both CERT levels and residual CERT activity through its phosphorylation, a condition that reduces the capacity of CERT to bind ceramide and prevent its localization to the Golgi apparatus.

### Effect of palmitate and glucose on [^3^H]Sph metabolism in INS-1 cells silenced for CERT

**Figure 6:**
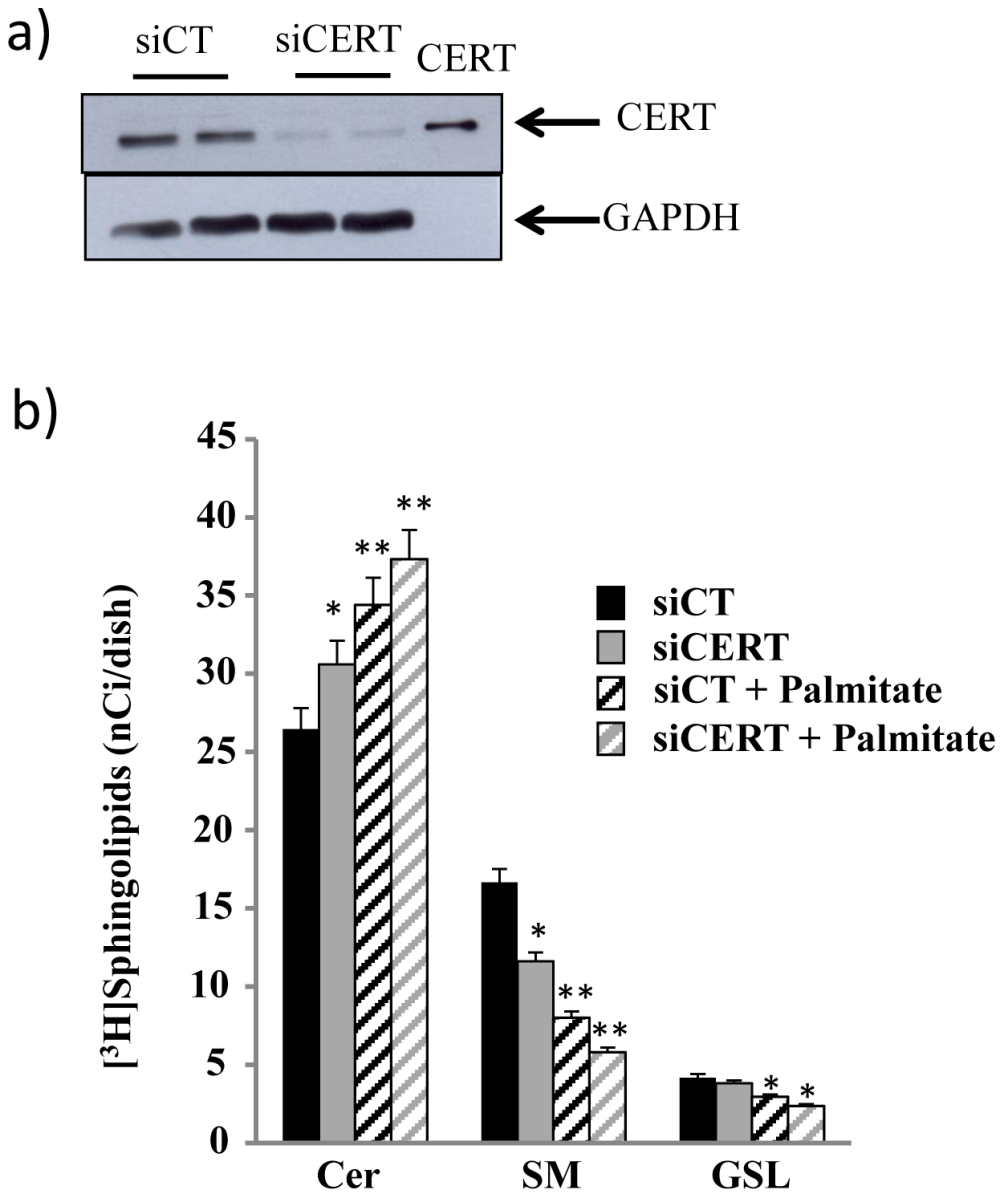
Palmitate and glucose affect vesicular-mediated Cer transport. a) Cells were transfected with a mix of S87 and S522 siRNA for CERT (siCERT) and the corresponding non-targeting corresponding sequences as control (siCT) and harvested in lysis buffer 72 h after transfection. 40 µg of protein from homogenate fractions and 2.4 ng of recombinant CERT (CERT) were analyzed by immunoblotting with polyclonal antibody anti-CERT and monoclonal anti-GAPDH. b) INS-1 cells down-regulated for CERT were treated for 12 h with or without palmitate in the presence of 30 mM glucose. Then the cells were pulsed with 0.3 µCi/ml [C3-^3^H]sphingosine for 1 h and processed and analyzed as described in the legend of Fig. 1. Data are mean ± S.D. of at least three independent experiments. *p<0.05 for siCERT compared with siCT and in GSL for siCT+palmitate vs siCT and siCERT+palmitate vs siCERT; **p<0.01 for siCT+palmitate compared with siCT and for siCERT+palmitate compared with siCERT.

On the basis of the results obtained, we next evaluated if CERT silencing could mimic the defect in Cer utilization induced by glucolipotoxicity. We examined the effects of palmitate with 30 mM glucose on [^3^H]Sph metabolism in control and CERT-down-regulated INS-1 cells. We set up optimal conditions to silence CERT and showed in the western blots that a **≥**90% reduction in CERT expression was achieved when cells were transfected with a mixture of S87 and S522 (1∶1 ratio) (Fig. 6a). As expected, down regulation of CERT promoted a significant but not complete reduction of Cer conversion to SM without modifying the amount of synthesized GSLs (Fig. 6b). Palmitate together with 30 mM glucose decreased Cer utilization for the synthesis of SM and GSLs in control cells (siCT) but also in siCERT ones (Fig. 6b). In particular, our results demonstrated that glucolipotoxicity induced a 52% decrease in [^3^H]SM in siCT cells and 65% in siCERT cells compared to cells treated with 30 mM glucose in the absence of palmitate. These results suggest that glucolipotoxicity, in addition to its effect on CERT, can also affect vesicular-mediated Cer transport in pancreatic β-cells.

### Effect of PI3K/Akt on [^3^H]Sph metabolism in INS-1 cells treated with palmitate and glucose

**Figure 7:**
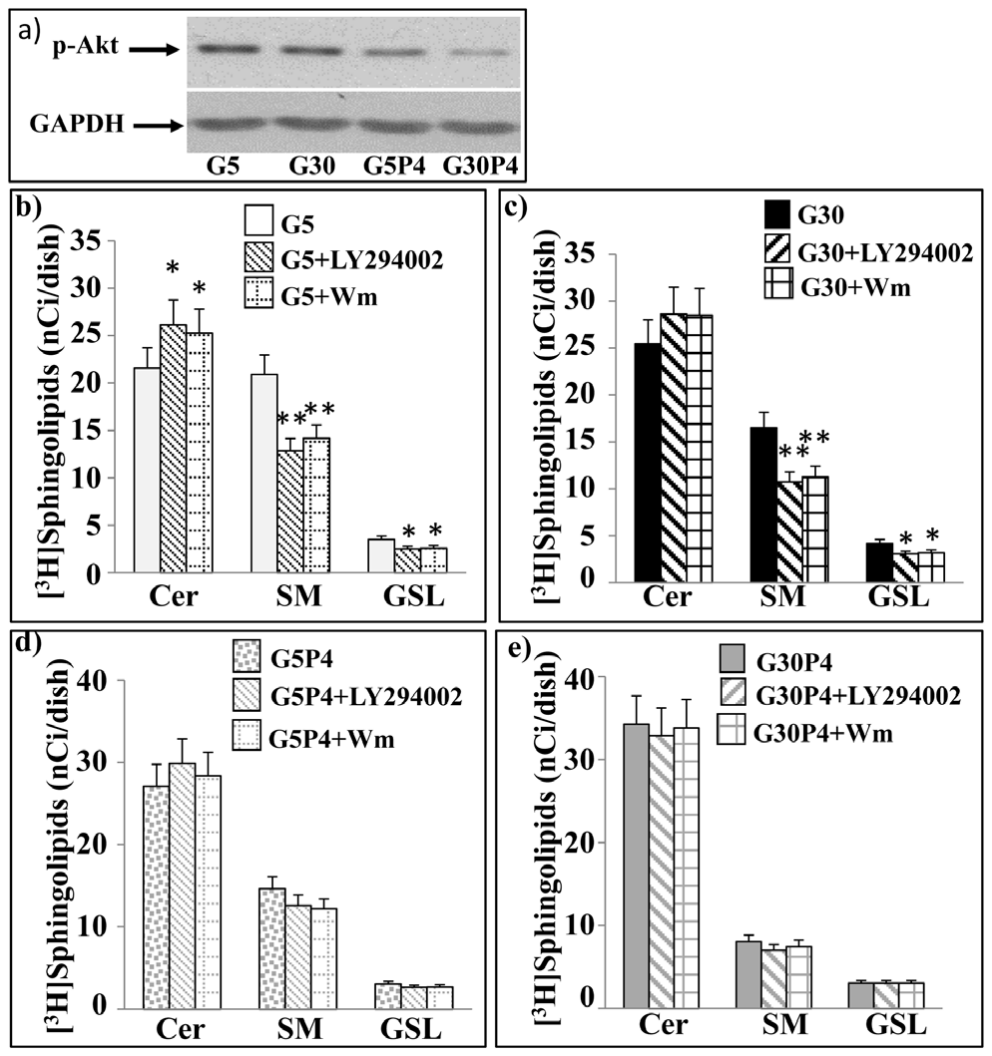
Palmitate and glucose inhibit vesicular-mediated Cer traffic through downregulation of PI3K/Akt pathway. **a**) INS-1 cells were treated with 5 mM or with 30 mM glucose ±0.4 mM palmitate and harvested for immunoblot analysis of phospho-Akt and GAPDH levels as described in experimental procedures. INS-1 cells were pretreated 30 min with or without 20 µM LY294002. The cells were then **b**) treated for 12 h with 5 mM glucose in the presence or absence of LY294002 or of Wm; **c**) treated for 12 h with 30 mM glucose in the presence or absence of LY294002 or Wm; **d**) treated for 12 h with 5 mM glucose plus 0.4 mM palmitate in the presence or absence of LY294002 or Wm; **e**) treated for 12 h with 30 mM glucose plus 0.4 mM palmitate in the presence or absence of LY294002 or Wm. Then cells were pulsed 1 h with [^3^H]Sph in the absence (*opened and dotted bars*) or presence of 20 µM LY294002 (*striped bars*) or 10 nM Wm (*square bars*). At the end of pulse, cells were harvested and submitted to lipid extraction and analyzed as described in the legend of Fig. 1. All values are the mean ± S.D. of at least three individual experiments. *p<0.05 for Cer and GSLs G5+LY294002 and G5+Wm compared with G5 and for GSLs G30+LY294002 and G30+Wm compared with G30; **p<0.01 for SM G5+LY294002 and G5+Wm compared with G5 and G30+LY294002 and G30+Wm compared with G30.

We previously demonstrated [38] that the PI3K/Akt pathway regulates the vesicular traffic of Cer in glioma cells, and we were interested in the present study to examine if this was also the case in INS-1 cells. We evaluated if a 12 h treatment with palmitate and high glucose levels was able to regulate the PI3K/Akt pathway in INS-1 cells. Western blot analysis with an antibody specific for Akt phosphorylated at Ser473, demonstrated that 30 mM glucose did not modify pAkt levels but 0.4 mM palmitate alone decreased pAkt levels and 0.4 mM palmitate together with 30 mM glucose induced a marked decrease of pAkt levels in INS-1 (Fig. 7a).

To investigate the effect of PI3K/Akt on Cer metabolism in INS-1 cells, we utilized LY294002 and Wm as pharmacological inhibitors of PI3K. We initially set up working concentrations to specifically inhibit PI3K. To do this, we evaluated the capacity of LY294002 and Wm to inhibit the PI3K/Akt pathway. Immunoblot analysis demonstrated that both 20 µM LY294002 and 10 nM Wm strongly reduced Akt activation (data not shown). INS-1 cells, treated with 5 mM or 30 mM glucose with or without palmitate, were pulsed with [^3^H]Sph in the presence or absence of LY294002 or Wm, and these last two molecules did not alter the [^3^H]Sph uptake, determined as the radioactivity measured in the total lipid extract (data not shown). In all cases, [^3^H]Sph was mainly metabolized to N-acylated compounds (Fig. 7b–e) and the extent of N-acylation (evaluated as the sum of tritiated Cer, SM and GSLs) was always very similar in the control and treated cells. However treatment with LY294002 or Wm strongly modified the radioactivity distribution among the different Sph metabolites both in the presence of 5 mM or 30 mM glucose (Fig. 7b and 7c). In 5 mM glucose+LY294002 or 5 mM glucose+Wm treated INS-1 cells, the radioactivity associated with [^3^H]Cer was 21% and 17% higher, respectively than that in 5 mM treated cells, with a concomitant reduction of both [^3^H]SM and [^3^H]GSL levels (39% and 29%, respectively in the presence of LY294002 and 32% and 27% respectively in the presence of Wm compared to 5 mM treated cells) (Fig. 7b). Similarly, in cells treated with 30 mM glucose, LY294002 and Wm promoted about 30% reductions in synthesized SM and 20% decrease in GSL compared to 30 mM glucose treated cells (Fig. 7c). Taken together these results suggest that the PI3K/Akt pathway can regulate the metabolic utilization of Cer through the modulation of the vesicular traffic of Cer, favouring the maintenance of low Cer levels in INS-1 cells even under conditions (high glucose) of reduced utilization of Cer for the biosynthesis of complex sphingolipids. In cells treated with 5 mM glucose+palmitate, LY294002 or Wm slightly reduced the amount of synthesized SM and GSL (Fig. 7d). In cells treated with high glucose in the presence of palmitate (characterized by a reduced utilization of Cer for the biosynthesis of complex sphingolipids as shown in Fig. 1), LY294002 or Wm did not further reduce the amount of synthesized SM and GSL (Fig. 7e). Altogether, these data suggest that palmitate inhibits vesicular-mediated Cer traffic through down-regulation of the PI3K/Akt pathway, and this effect is potentiated in glucolipotoxic conditions, thereby contributing to the accumulation of Cer in pancreatic β-cells.

## Discussion

The most relevant result obtained by this investigation is that in pancreatic β-cells, glucolipotoxicity impairs Cer traffic from the ER to the Golgi apparatus, thus promoting the accumulation of Cer in the ER. As a model for mimicking glucolipotoxicity, we incubated INS-1 cells with 0.4 mM palmitate in the presence of high glucose concentrations (30 mM). This condition resulted in a 60% reduction in cell viability after a 24 h treatment. Studies using [^3^H]Sph as a metabolic precursor showed that glucolipotoxicity strongly reduced the utilization of newly synthesized Cer mainly for the biosynthesis of SM and, to a lesser extent, for synthesis of GlcCer and complex GSLs. This occurred after a 12 h treatment, when all INS-1 cells are still viable but show a significant ER stress response (Fig. 1b). It is noteworthy that palmitate-induced reduction of Cer utilization for complex sphingolipid biosynthesis was strongly potentiated by high glucose levels. As a consequence, the observation of increased Cer levels is in agreement with previously published data [11], [35]. We show for the first time that this increase was associated with a significant reduction of total SM but not of GlcCer levels in INS-1 cells. Glucolipotoxic conditions does not significantly modify GlcCer mass level but this does not exclude that the amount of complex GSLs could be reduced. Moreover we cannot exclude that glucosylceramide synthase is increased by palmitate as Boslem and co-workers [20] have shown that palmitate increase GlcCer in MIN6 β-cells. Interestingly, glucolipotoxicity lowered only saturated SM species in β-cells, favouring the accumulation of saturated Cer species such as C18∶0-Cer, which are pro-apoptotic in β-cells [12]. The decrease in SM biosynthesis suggests that glucolipotoxicity could affect SM synthase activity and/or the availability of its substrate by inhibiting Cer traffic from the ER to the Golgi. However, the capacity of INS-1 β-cells to synthesize SM from a diffusible substrate such as NBD-Cer is maintained, indicating that SM synthase activity is preserved during glucolipotoxic conditions. The analysis of intracellular distribution of BODIPY-C_5_-Cer, which mimics the intracellular movements of naturally occuring Cer [26], [34], [39], provided evidence that glucolipotoxicity impairs the intracellular traffic of Cer from ER to the Golgi apparatus in INS-1 β-cells, supporting the idea that, under glucolipotoxic conditions, this mechanism can contribute to the accumulation of Cer at the ER. Recent studies have shown that specific accumulation of Cer at the ER due to palmitate can induce ER stress and apoptosis of pancreatic β-cells [14], [19], [20], [34]. This phenomenon could be alleviated by over-expression of glucosylceramide synthase in β-cells, an enzyme which transforms ceramide into glucosylceramide, thereby preventing excessive accumulation of ceramide in the ER [20]. Importantly, our results enforce the crucial role of Cer accumulation at the ER in induced apoptosis and the loss of β-cell mass that contributes to the development of type II diabetes. Moreover, our results confirmed that glucolipotoxicity increased total S1P levels in INS-1 cells in agreement with Véret and co-workers [35] who showed that palmitate treatment not only induce anti-apoptotic signals but also pro-apoptotic signals such as the production of S1P. Evidence from published literature [29] demonstrated that the decrease in S1P levels is associated with Cer traffic impairment, leading us to hypothesize that increased S1P levels are unlikely to be involved in the initiation of defective ceramide transport. Additionally, the increased in S1P levels are also unlikely to be sufficient to restore normal ceramide trafficking in order to avoid ceramide accumulation to counteract the effect of glucolipotoxicity.

Two main mechanisms are involved in ceramide transport from the ER to the Golgi apparatus: a protein-mediated transport that is mediated by CERT [23], [24], and a CERT-independent vesicular transport pathway [23], [26]; our data demonstrate that both of these pathways are strongly inhibited by glucolipotoxicity. In relation to CERT, a 12 h treatment of β-cells with glucolipotoxic conditions but not the treatment with high glucose and palmitate separately, induced a significant decrease in the total amount of the CERT protein and steady-state transcript levels, suggesting a reduced rate of CERT protein synthesis in glucolipotoxic conditions. In this respect, Granero et al. identified a human-specific TNFα-responsive promoter for CERT [40] as a possible mechanism of transcriptional CERT regulation. However, we cannot exclude the possibility that, during glucolipotoxic stress, CERT could also be cleaved by caspase-3, an enzyme highly activated by glucolipotoxicity in β-cells [12] as has been proposed in Hela cells under pro-apoptotic stress [41]. Moreover we also found that, under glucolipotoxic conditions, residual CERT protein is highly phosphorylated and is no longer able to localize at the Golgi apparatus in INS-1 cells. CERT specifically targets the Golgi apparatus through its PH domain, which selectively recognizes phosphatidyl-inositol-4-phosphate (PI-4P) at the Golgi, and its hyperphosphorylation results in the repression of PI4P binding activity of the PH domain [37]. Palmitate or glucose alone did not modify either phosphorylation or CERT localization. However, a recent paper [36] demonstrated that short term exposure (3h) to palmitate increased phosphorylation of CERT in INS-1 cells and this was associated with the inhibition of insulin gene expression. This appears to suggest that acute and chronic exposure to palmitate might differentially affect CERT activity. Overall, glucolipotoxicity strongly decreases the amount of active CERT and the amount of remaining CERT is mostly inactive. Altogether, these data demonstrate that glucolipotoxicity impairs the CERT-mediated Cer traffic from the ER to the Golgi apparatus, thus supporting the idea that, under glucolipotoxic conditions, this mechanism contributes to the accumulation of Cer at the ER. Therefore, CERT is important not only in the regulation of the insulin gene expression [36], but also in the predisposition to β-cell death.

The vesicle-mediated pathway of Cer with the transport of a thousand lipid molecules per transfer step could provide a feasible mechanism to compensate for CERT defect in the bulk of SM biosynthesis. We demonstrated that in INS-1 cells where expression of CERT is silenced (a condition able to mimic the effect of glucolipotoxicity on CERT), glucolipotoxic conditions are still able to further decrease the metabolic utilization of Cer for the synthesis of SM and GSLs, lending credence that vesicular traffic of Cer is impaired. Additionally, we observed in INS-1 cells that the PI3K/Akt pathway regulates Cer metabolism by controlling the vesicular transport of Cer between the ER and the Golgi apparatus, similar to glioma cells [38]. Glucolipotoxic conditions promote a strong inhibition of the PI3K/Akt pathway, consistent with previous studies showing that long-term exposure to glucolipotoxic conditions and/or high palmitate decreases the PI3K/Akt pathway in pancreatic β-cells [42]–[44]. Our experiments also showed that palmitate alone partially decreased pAkt levels and this was associated with the inhibition of Cer vesicular traffic. The role of the PI3K/Akt pathway in mediating the effects of palmitate and glucolipotoxicity on Cer vesicular flow is confirmed by the evidence that the PI3K inhibitors, LY294002 and Wm, which are not effective in glucolipotoxic conditions, have a slight effect on Cer flow in cells treated with palmitate in the presence of 5 mM glucose, probably because palmitate alone only partially inhibits pAkt. It is worth noting that the reduced Cer utilization is higher than the simple sum of the effects induced by the two single nutrients separately. The results of our study support the idea that glucolipotoxicity inhibits the PI3K/Akt pathway in pancreatic β-cells, which in turn inhibits the vesicular trafficking of Cer. On the other hand, 30 mM glucose decreased the biosynthesis of SM but does not modify pAkt levels and Cer traffic suggesting that this effect on SM could be associated with increased N-SMase activity, consistent with previous papers [18], [19] demonstrating that high glucose levels increased N-SMase activity.

**Figure 8:**
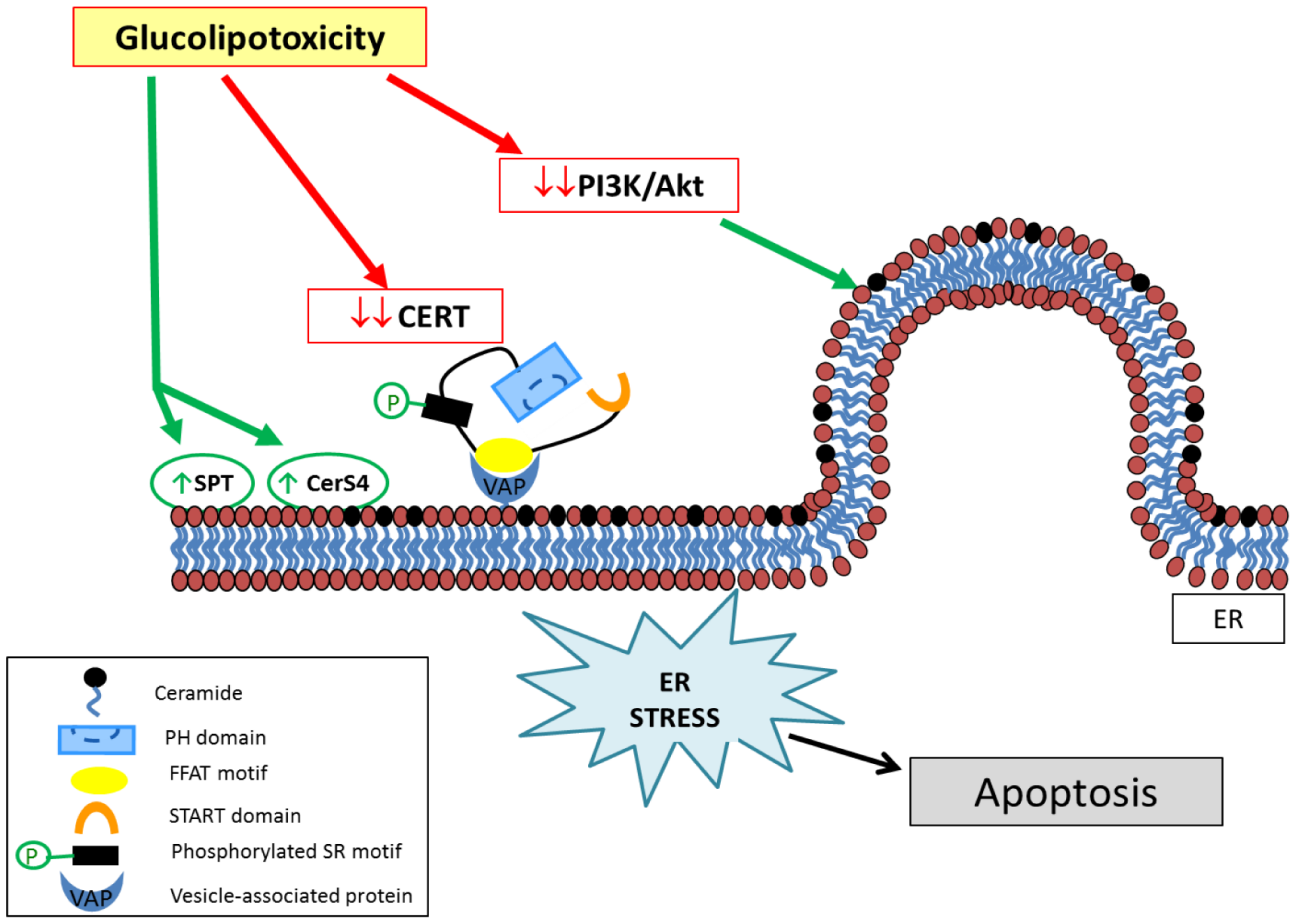
Schematic representation of the model showing the involvement of ceramide traffic in ER stress induced by glucolipotoxicity. Glucolipotoxicity impairs CERT- and vesicular-mediated Cer traffic. Glucolipotoxicity decrease the amount of active CERT significantly decreasing a) the total amount of the protein and b) the phosphorylation of CERT SR motif that is no longer able to localize at the Golgi apparatus. Moreover glucolipotoxicity inhibits PI3K/Akt pathway that could in turn impairs vesicular trafficking of Cer from the ER to the Golgi apparatus. Both transport systems contribute to the accumulation of Cer at the ER, thereby inducing ER stress. Furthermore ceramide synthase 4 (CerS4) [12] and serine palmitoyltransferase (SPT) [16], [17], both residing in the endoplasmic reticulum (ER), have been shown to be involved in regulating Cer levels in β-cells in response to lipotoxicity and/or glucolipotoxicity.

Altogether, our data demonstrate that the CERT- and vesicular-mediated Cer trafficking pathways can separately contribute to the control of sphingolipid metabolism and Cer levels in INS-1 cells, thus participating in regulating the accumulation of ER-associated Cer involved in the regulation of pancreatic β-cell function and death during type II diabetes [14], [20]. In support to this new idea, Guo and co-workers recently showed that down-regulation of CERT by specific siRNA potentiated palmitate-induced inhibition of insulin gene expression in pancreatic β-cells [36]. Moreover, these findings suggest that an impairment in ER to Golgi Cer traffic can act synergistically, leading to enhanced *de novo* Cer biosynthesis [12], [16], resulting in accumulation of Cer in the ER in response to glucolipotoxicity (Fig. 8).

Further understanding of the mechanisms that regulate the accumulation of Cer at the ER will be important for developing new strategies to prevent type II diabetes. Moreover, the capacity of the PI3K/Akt pathway to regulate sphingolipid metabolism may also be pathologically relevant in β-cells if we consider that the PI3K/Akt pathway plays a crucial role in the control of β-cell mass and function by modulating a dynamic balance of proliferation, cell size and apoptosis [45].

## Acknowledgments

We thank Dr. Maria Antonietta De Matteis, for the CERT-GFP plasmid, and Dr. Suhas Shinde for PL analysis.
